# Incidence and Burden of Respiratory Syncytial Virus-Associated Hospitalizations Among People 65 and Older in France: A National Hospital Database Study

**DOI:** 10.1093/ofid/ofaf528

**Published:** 2025-09-15

**Authors:** Stéphane Marot, Clarisse Demont, Théophile Cocherie, Miao Jiang, Charlotte Charpentier, Andre Araujo, Tianyi Lu, Mathieu Uhart, Nadia El Mouaddin, Magali Lemaitre, Sophie Larrieu, Clélia Bignon-Favary, Emilie Lambourg, Arnaud Cheret, David Martin, Anne-Geneviève Marcelin, Diane Descamps, Vincent Calvez, Quentin Le Hingrat

**Affiliations:** Sorbonne Université, INSERM, Institut Pierre Louis D'Epidémiologie et de Santé Publique (IPLESP), AP-HP, Hôpital Pitié-Salpêtrière, Service de Virologie, Paris, France; Moderna, Inc., Paris, France; Sorbonne Université, INSERM, Institut Pierre Louis D'Epidémiologie et de Santé Publique (IPLESP), AP-HP, Hôpital Pitié-Salpêtrière, Service de Virologie, Paris, France; Moderna, Inc., Cambridge, Massachusetts, USA; Service de Virologie, Université Paris Cité, INSERM, IAME, UMR 1137, AP-HP, Hôpital Bichat Claude-Bernard, Paris, France; Moderna, Inc., Cambridge, Massachusetts, USA; Moderna, Inc., Cambridge, Massachusetts, USA; Moderna, Inc., Paris, France; Moderna, Inc., Paris, France; Horiana, Bordeaux, France; Horiana, Bordeaux, France; Horiana, Bordeaux, France; Horiana, Bordeaux, France; Moderna, Inc., Paris, France; Moderna, Inc., Cambridge, Massachusetts, USA; Sorbonne Université, INSERM, Institut Pierre Louis D'Epidémiologie et de Santé Publique (IPLESP), AP-HP, Hôpital Pitié-Salpêtrière, Service de Virologie, Paris, France; Service de Virologie, Université Paris Cité, INSERM, IAME, UMR 1137, AP-HP, Hôpital Bichat Claude-Bernard, Paris, France; Sorbonne Université, INSERM, Institut Pierre Louis D'Epidémiologie et de Santé Publique (IPLESP), AP-HP, Hôpital Pitié-Salpêtrière, Service de Virologie, Paris, France; Service de Virologie, Université Paris Cité, INSERM, IAME, UMR 1137, AP-HP, Hôpital Bichat Claude-Bernard, Paris, France

**Keywords:** respiratory syncitial virus, burden, elderly and comorbid population, healthcare resources

## Abstract

**Background:**

Respiratory Syncytial Virus (RSV) presents a serious threat to older adults, particularly those with chronic conditions, and may lead to severe issues. Hospitalizations are frequently underreported due to diagnostic challenges and a lack of standardized testing. This study estimates national-level RSV hospitalization rates and examines the clinical and economic burden in high-risk elderly populations in France.

**Method:**

RSV-coded hospitalizations (2017–2022) were identified using the French National Hospital Discharge database. A correction factor, derived from virological data from two local hospitals, was applied to adjust for under-reporting. Incidence rates were calculated using demographic data, focusing on adults aged 75+ and high-risk individuals aged 65–74 with comorbidities. Hospitalization characteristics and costs were also analyzed.

**Results:**

For adults aged 75+, the adjusted incidence of RSV hospitalizations ranged from 85 to 221 per 100,000. Inpatient mortality was 8.9%–10.4%, and annual adjusted costs ranged from €27 to €76 million, with intensive care units (ICU) admissions contributing heavily. High-risk adults aged 65–74 had higher adjusted incidence rates (161–735 per 100,000), along with increased ICU admission rates and disproportionately higher costs due to intensive care needs.

**Conclusions:**

The significant burden of RSV on adults aged 75+ and high-risk adults aged 65–74 with chronic conditions remains underreported. Improved diagnostics and targeted vaccination programs are essential to reduce hospitalizations, mortality, and healthcare costs in these vulnerable groups.

## Teaser

This study highlights the high burden of RSV hospitalizations among older adults in France, emphasizing high incidence, mortality, and costs, underscoring the need for improved diagnostics, targeted vaccination, and policies to enhance prevention and care for vulnerable populations.

Respiratory Syncytial Virus (RSV) is a respiratory virus that causes excessive morbidity and mortality in certain populations: infants, older adults, and adults with chronic conditions like chronic obstructive pulmonary disease (COPD), cardiovascular diseases, or diabetes [1–14]. In older adults, RSV can cause severe respiratory infections, decompensation of underlying diseases, and lead to death [15–17]. Age-related declines in immunity further increase susceptibility [9].

RSV-related hospitalizations in older adults are often underreported due to several factors. Symptoms of RSV are similar to those of other respiratory infections, complicating clinical diagnosis without lab confirmation. Until recently, the lack of standardized RSV testing guidelines for adults, particularly in primary care and hospital settings, led to inconsistent screening [18, 19]. Additionally, severe RSV cases may be misclassified due to secondary infections or exacerbation of chronic conditions [7, 20, 21].

In metropolitan France, RSV typically circulates from mid-October to mid-January, similar to other European countries with temperate climates [22–24]. Under-reporting of RSV cases, including outpatient visits and hospitalizations, was acknowledged in recent vaccine recommendations by the French Health Authority (HAS), which now advises RSV vaccination for adults aged 75+ and those aged 65+ with respiratory or cardiac conditions [24]. Although there is no etiologic treatment for RSV, new vaccines have been recommended to mitigate its impact.

Modeling approaches have been used to assess RSV burden in older adults in high-income countries, confirming substantial disease burden in this population [7, 12]. French data have been analyzed using similar methods, but the lack of virological data has limited their accuracy [25]. The French National Hospital Discharge Database (PMSI) provides comprehensive hospitalization data, but adding real-world virological data should improve the estimation of the true burden of RSV [5, 22].

By combining data from the PMSI with virological data, this study has two aims (1) to estimate the annual incidence of RSV-related hospitalizations in France from 2017 to 2022 among adults aged 75+ and those aged 65–74 with chronic conditions, and (2) to assess the clinical impact, length of hospital stay, and economic burden associated with these hospitalizations.

## METHODS

### Study Design

This is an annual cross-sectional study conducted from all RSV-coded hospitalizations reported in the claim database among persons aged 65 and older in France. The study examined five seasons from July 2017 to June 2022, with an additional analysis including data from July to December 2022.

### Data Sources

Hospitalization data were obtained from the PMSI, a national claim database covering all hospitalizations in France's public and private healthcare centers. Specific hospital data are retrievable from the PMSI. The data includes patient demographics, discharge details, length and cost of stays, International Classification of Diseases (ICD)-10-coded diagnoses, and medical procedures. Each patient is assigned an anonymous ID consistent across years. PMSI, part of the French National Health Data System (SNDS), is recommended by the French National Authority for Health (HAS) for real-world studies in the hospital setting due to its comprehensive nature and low missing data rate [26].

Virological dataset was created from data collected at Pitié-Salpêtrière and Bichat Claude Bernard University hospitals, where all patients with symptoms of a respiratory infection were systematically tested with multiplex PCR assays (BioFire® FilmArray Respiratory Panel and QIAstat-Dx Respiratory panel). Both systems present high detection accuracy (Positive Percent Agreement > 95% and Negative Percent Agreement > 97%) [27, 28]. No individual link was made between the PMSI and the virological dataset.

The number of adult populations in France for the study period was sourced from the French National Institute for Statistics and Economic Studies (INSEE). Population denominator from Pitié-Salpêtrière and Bichat Claude Bernard University hospitals was not available.

### Study Population

**Figure 1.**
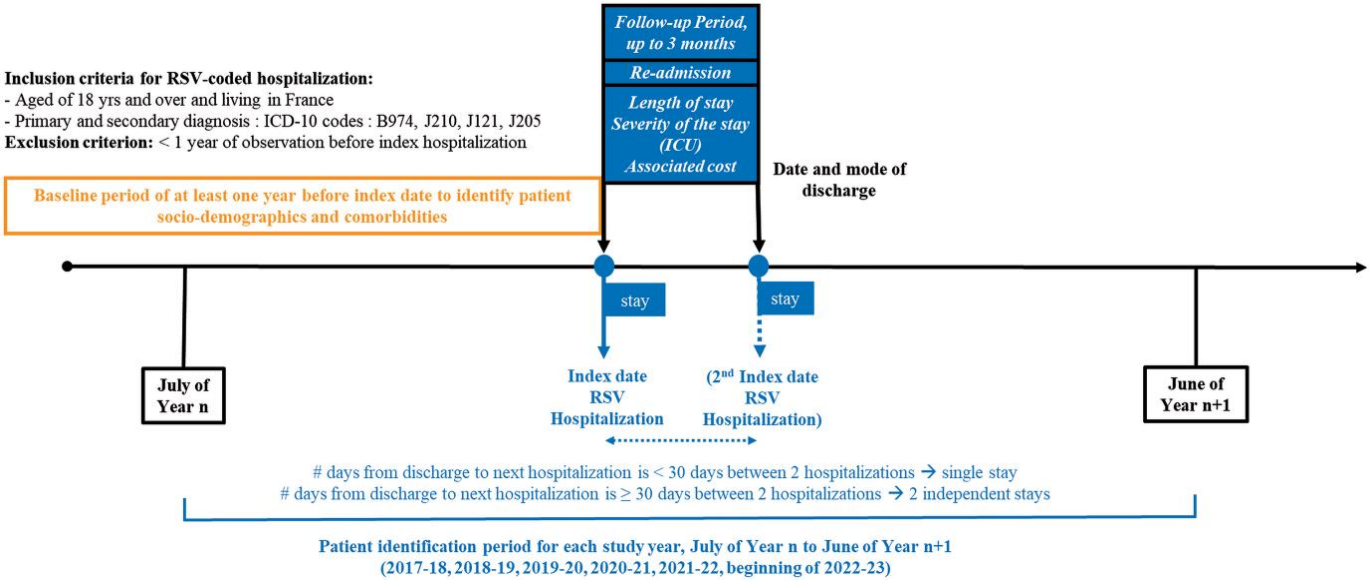
Study Design Diagram. Patient identification period for each study year, July of Year *n* to June of Year *n* + 1 (2017–2018, 2018–2019, 2019–2020, 2020–2021, 2021–2022, July–Dec 2022).

Each season, patients aged 65 and older with RSV-coded hospitalizations (Index Hospitalizations) defined by ICD-10 codes for primary/related or secondary diagnoses: B974 (RSV as the cause of diseases classified in other chapters), J210 (Acute bronchiolitis due to RSV), J121 (Pneumonia due to RSV), and J205 (Bronchitis due to RSV) were included. Patient characteristics were assessed one year at baseline and up to 3 months of post-index follow-up. Unique hospitalizations were identified as those separated by more than 30 days (Figure 1). Patients with less than 1 year of medical observation before their index hospitalization were excluded.

Two groups were defined based on French HAS RSV vaccine guidelines [24]:

1. Population 75+: Adults aged 75 and older.
2. High-Risk Population 65–74: Adults aged 65–74 with a prior-year hospitalization for COPD (J44.0, J44.1) or Congestive Heart Failure (CHF) (I50-).

### Statistical Methods

#### Calculation of the RSV Correction Factor (CFRSV)

RSV-correction factor (CFRSV) was calculated as the ratio of two numbers: the numerator as the number of laboratory-confirmed RSV cases reported from the virological dataset of the two hospitals; the denominator as the number of RSV-coded hospitalizations reported in the PMSI from the same two hospitals. These numbers were reported by study season, age group, and care unit (intensive care units (ICU) vs. Non-ICU). When sample sizes were less than 10, data were combined at appropriate levels to maintain anonymization, and CFRSV was calculated at the corresponding aggregated level. The higher the CFRSV, the higher the extent of under-reporting.

#### Number and Incidence of RSV Hospitalizations

The unadjusted RSV-coded hospitalizations at the national level were reported from PMSI by study season, age group, and care unit (ICU vs. non-ICU). To overcome under-reporting of RSV-coded hospitalizations in the PMSI database, the CFRSV derived above was multiplied by the unadjusted RSV-coded hospitalizations to estimate the adjusted number of RSV hospitalizations by season, age group, and care unit at the national level.

**Figure 2.**
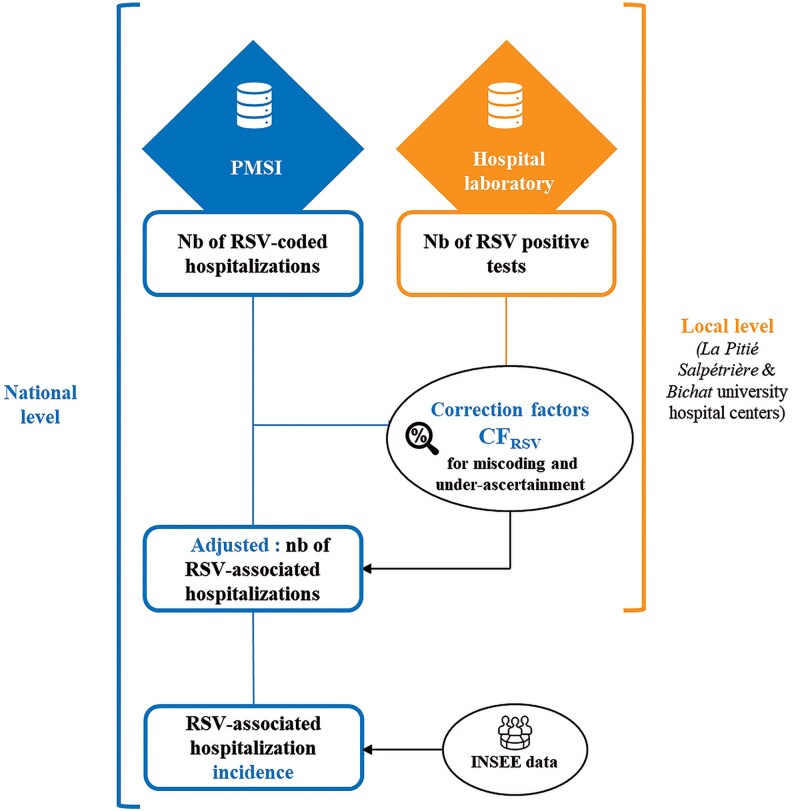
Statistical approach for incidence calculation.

Incidence rates at the national level, both unadjusted RSV-coded hospitalizations and adjusted RSV hospitalizations (RSV-coded hospitalizations corrected by the CFRSV), were calculated by dividing these hospitalization numbers by the number of adult populations in France (based on INSEE data) and expressed per 100,000 residents (Figure 2). Since INSEE and the National Health Insurance (CNAM) do not provide COPD-specific data, the incidence was calculated using the broader Chronic Respiratory Disease (CRD) category. (More details are provided at https://data.ameli.fr/pages/pathologies/?refine.patho_niv1=Maladies%20respiratoires%20chroniques%20(hors%20mucoviscidose)) Results were reported for three groups: adults aged 75+, adults aged 65–74 with CHF, and adults aged 65–74 with CRD.

We also conducted a sensitivity analysis that broadened the case definition to include hospitalizations with cardiorespiratory (CR) primary diagnoses (I21, I50, I63, I64, and J00-J99) to explore potential under-reporting of RSV cases (Supplementary materials provide more details on this sensitivity analysis).

#### Characteristics of RSV-coded Hospitalizations

A descriptive analysis was conducted on unadjusted RSV-coded hospitalizations identified in the PMSI database per study season. Characteristics included length of stay (LOS), need for intensive care or mechanical ventilation, and inpatient mortality. Additionally, 90-day hospital readmissions were analyzed for any cause, respiratory (ICD-10 J00-J99), or cardiorespiratory causes (ICD-10 I00-I99), as well as inpatient mortality within 90 days post-discharge.

#### Cost of RSV Hospitalization

Costs were estimated using the PMSI database, which categorizes hospital stays into GHS (Groupe Homogène de Séjour), comparable to Diagnosis-Related Groups (DRGs). Additional costs were calculated based on procedures coded for intensive care, continuous monitoring, and resuscitation.

In this study, hospitalization costs were calculated per patient per season, with further breakdowns by ICU usage and GHS categories, converted to 2022 Euros.

National RSV costs were estimated by multiplying the average cost per stay, specific to whether there was a stay in ICU or not, by the number of unadjusted RSV-coded hospitalizations (with or without ICU), then multiplying the correction factor CFRSV. It was assumed that costs per stay remained consistent between unadjusted RSV-coded hospitalizations and adjusted RSV hospitalizations, a common approach in PMSI-based research [29, 30]. Mean or median costs were presented depending on the distribution.

Statistical analyses were performed using the SAS software for Windows (Version 9.4), with results stratified by study season and the population group.

## RESULTS

### Study Population

Within the PMSI database, 353 patients with unadjusted RSV-coded hospitalizations were identified at Pitié-Salpêtrière and Bichat Claude Bernard University hospitals from July 2017 to December 2022. Among them, 54.1% were aged 65 and older, 52.1% female, and 28.3% had at least one ICU stay. Compared to the patients at the national level with unadjusted RSV-coded hospitalizations, patients from the two hospitals were younger, with a slightly higher ICU stay percentage (Supplementary Table 1).

### Correction Factor

From the virological dataset, between the 2017–2018 and 2021–2022 seasons, there was a slight increase in the total number of RSV tests performed across the two hospitals, though the number of confirmed positive RSV cases declined. Consequently, the percentage of positive RSV tests dropped from approximately 4% in 2017–2018 to 0.9% in 2021–2022, with a slight recovery in July–December 2022 (3%).

Due to the small sample size in each age category and care unit, the CFRSV was calculated using combined data for all patients aged 65 and older across ICU and non-ICU units. Take the 2018–2019 season, for example, there were 282 positive RSV tests reported from the virological data, and 58 unadjusted RSV-coded hospitalizations for the two hospitals reported in the PMSI, corresponding to a CFRSV of 4.86. Across seasons, the CFRSV ranged from 4.00 to 5.22, indicating that for every 4–5 virologically confirmed RSV hospitalizations, only 1 RSV-coded hospitalization was recorded in the PMSI database, reflecting 75%–80% under-reporting. Data from the 2020-21 season were excluded due to limited sample size (Table 1).

**Table 1.** Calculation of the correction factors (CFRSV)^a^ by each season^b^ among those aged 65 and older (all units included).

Age 65 And Older	2017–2018	2018–2019	2019–2020	2020–2021^c^	2021–2022	July–Dec 22
Total number of RSV tests in Pitié/Bichat University hospitals	5,280	7,498	8,024	–	9,366	3,701
Number of positive RSV tests in Pitié/Bichat University hospitals	214	282	189	–	84	114
% of positive RSV tests in LaPitié/Bichat University hospitals	4.1%	3.8%	2.4%	–	0.9%	3.1%
Number of RSV-coded hospitalizations at Pitié/Bichat University hospitals from PMSI	41	58	39	–	21	31
Correction factor (CFRSV)	**5.22**	**4.86**	**4.85**	**–**	**4.00**	**3.68**

^a^Correction factor (CFRSV)= Number of RSV positive tests reported from the virological dataset of Pitié-Salpêtrière and Bichat Claude Bernard University hospitals divided by the number of RSV coded-hospitalizations in the PMSI from these two hospitals.

^b^Each study season is July of the current year to June next year.

^c^CFRSV was not calculated for 2020–2021 season due to a small sample size of less than 10.

### Number and Incidence of RSV Hospitalizations at the National Level

**Figure 3.**
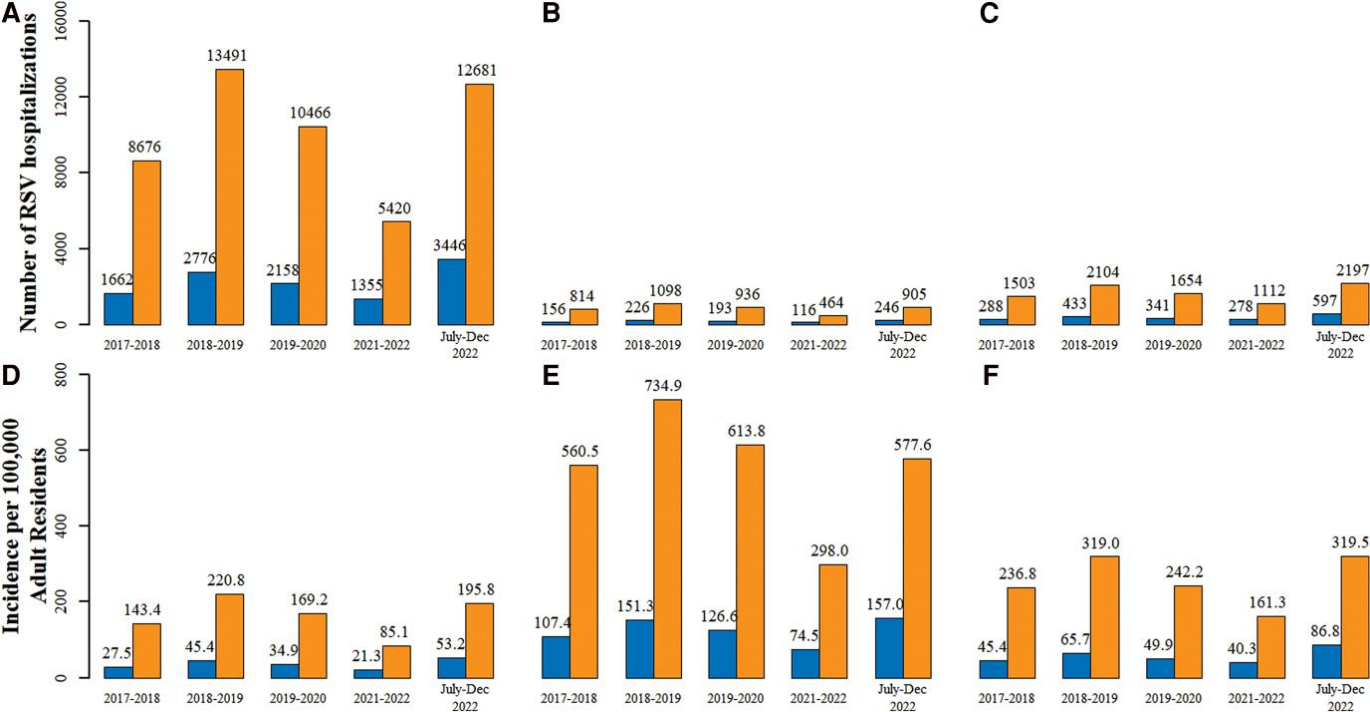
Legend: Number of RSV hospitalizations among patients (*A*) aged 75 and over, (*B*) aged 65–74 with CHF, and (*C*) aged 65–74 with CRD; and incidence per 100,000 adult residents among patients (*D*) aged 75 and over, (*E*) aged 65–74 with CHF, and (*F*) aged 65–74 with CRD. Blue bars represent the number and incidence reported from PMSI, and orange bars represent the number and incidence adjusted by CFRSV.

Figure 3 presents the unadjusted number and incidence of RSV hospitalizations as reported by PMSI, as well as the CFRSV-adjusted numbers (unadjusted number multiplying the corresponding CFRSV). For individuals aged 75 and older, the adjusted estimated annual incidence of RSV hospitalizations varied between 85 and 221 per 100,000 from 2017 to 2018 to the first half of 2022–2023. The lowest incidence was 85 per 100,000 in 2021–2022, while the highest reached 221 per 100,000 in 2018–2019, corresponding to a number of 5,420–13,491 RSV-adjusted hospitalizations. Among those aged 65-74 with comorbidities, the adjusted incidence was higher, ranging from 161 to 319 per 100,000 for those with CRD, and 298 to 735 per 100,000 for those with CHF, corresponding to a number of 1,112–2,197 RSV-adjusted hospitalizations for those with CRD and 464–1,098 RSV-adjusted hospitalizations for those with CHF, respectively (Figure 3).

Consistent with these estimates, Supplementary Table 2 shows that after applying the CFRSV, the total number of RSV hospitalizations among all adults aged ≥ 65 years in France ranged from 7,608 to 17,831. Using the broader cardiorespiratory definition among all adults aged ≥ 65 years, the estimated number of RSV hospitalizations rose to between 9,363 and 24,527 (Supplementary Table 3), highlighting additional under-detection in older adults.

### Characteristics of Unadjusted RSV-coded Hospitalizations

Table 2 presents the characteristics of unadjusted RSV-coded hospitalizations reported from PMSI. Due to limited sample sizes in 2020-21 and incomplete data for 2022-23 (through December 2022), the analysis focuses on three pre-COVID-19 pandemic seasons (2017-18, 2018-19, 2019-20) and one post-pandemic season (2021-22).

**Table 2.** Description of unadjusted RSV-coded Hospitalizations Reported from PMSI.

	2017–2018	2018–2019	2019–2020	2020–2021^b^	20212022
Age ≥75 years					
Total number of RSV hospitalization coded	**1,662**	**2,776**	**2,158**	**–**	**1,355**
Mean (SD) length of stay (LOS) of RSV hospitalization (in days)	12.1 (10.1)	12.5 (10.8)	12.9 (12.7)	**–**	12.2 (10.0)
At least one stay in ICU (intensive or continuous care or resuscitation), *N* (%)	307 (18.5)	507 (18.3)	395 (18.3)	**–**	164 (12.1)
Mean (SD) LOS in intensive or continuous care or resuscitation (in days)	6.8 (8.3)	7.1 (8.0)	6.9 (7.3)	**–**	7.0 (6.4)
Death during the RSV hospitalization, *N* (%)	150 (9.0)	271 (9.8)	191 (8.9)	**–**	141 (10.4)
Patients surviving the index hospitalizations	**1,512**	**2,505**	**1,967**	**–**	**1,214**
All-cause rehospitalization within 3 months^a^, *N* (%)	570 (37.7)	877 (35.0)	659 (33.5)	**–**	419 (34.5)
At least one cardio-respiratory rehospitalization within 3 months (DP: I21, I50, I63, I64, J00-J99)^a^, *N* (%)	242 (16.0)	327 (13.1)	223 (11.3)	**–**	145 (11.9)
At least one respiratory rehospitalization within 3 months (DP: J00-J99)^a^, N (%)	149 (9.9)	221 (8.8)	135 (6.9)	**–**	82 (6.8)
Hospital death within 3 months^a^, *N* (%)	66 (4.4)	105 (4.2)	107 (5.4)	**–**	54 (4.4)
**Age 65-74, with chronic conditions**					
Total number of RSV hospitalization coded	**274**	**415**	**321**	**–**	**248**
Mean (SD) LOS of RSV hospitalization (in days)	13.4 (14.4)	13.4 (13.2)	14.3 (12.8)	**–**	13.3 (13.4)
At least one stay in ICU (intensive or continuous care or resuscitation), *N* (%)	138 (50.4)	189 (45.5)	157 (48.9)	**–**	107 (43.1)
Mean (SD) LOS of stay in intensive or continuous care or resuscitation (in days)	9.6 (12.3)	8.2 (11.7)	9.7 (9.5)	**–**	9.0 (10.9)
Death during the RSV hospitalization, *N* (%)	22 (8.0)	25 (6.0)	29 (9.0)	**–**	21 (8.5)
Patients surviving the index hospitalizations	**252**	**390**	**292**	**–**	**227**
All-cause rehospitalization within 3 months^a^, *N* (%)	119 (47.2)	176 (45.1)	130 (44.5)	**–**	103 (45.4)
At least one cardio-respiratory rehospitalization within 3 months (DP: I21, I50, I63, I64, J00-J99) ^a^, *N* (%)	57 (22.6)	65 (16.7)	54 (18.5)	**–**	35 (15.4)
At least one respiratory rehospitalization within 3 months (DP: J00-J99) ^a^, N (%)	48 (19.0)	53 (13.6)	36 (12.3)	**–**	29 (12.8)
Hospital death within 3 months^a^, *N* (%)	≤ 10	12 (3.1)	15 (5.1)	**–**	≤ 10

Abbreviation: DP, primary diagnosis code.

^a^Use of calendar months for years before 2020, and with the whole date for years after 2020.

^b^Season 2020–2021 was not reported due to a small sample size of less than 10.

For patients aged 75 and older, the mean LOS ranged between 12.1 and 12.9 days, with 12.1% to 18.5% requiring ICU care, where the average ICU stay was between 6.8 and 7.1 days across the seasons. Inpatient mortality ranged from 8.9% to 10.4%, and over one-third (33.5%–37.7%) were re-admitted within 3 months, primarily for respiratory (6.8%–9.9%) or cardio-respiratory causes (11.3%–16.0%). Inpatient mortality within 3 months post-discharge ranged from 4.2% to 5.4% (Table 2).

Patients aged 65–74 with chronic conditions conditions, including COPD and/or CHF, had a longer LOS (13.3–14.3 days) and higher ICU admission rates (43.1%–50.4%) with an average ICU stay of 8.2–9.7 days. This group had higher re-admission rates (44.5%–47.2%) for respiratory (12.3%–19.0%) and cardio-respiratory causes (15.4%–22.6%). Inpatient mortality was slightly lower, but post-discharge deaths were too few to be reported (Table 2).

### Cost of RSV Hospitalization

Across the seasons, for patients aged 75 and older, the average cost per unadjusted RSV-coded hospitalization varied from €5,618 in 2017–2018 to €5,021 in 2021–2022, peaking at €5,706 in 2019–2020. Unadjusted RSV-coded hospitalizations with ICU admissions were more costly, spanning from €10,482 in 2017–2018 to €9,536 in 2021–2022, with a peak of €11,127 in 2019–2020. Due to small sample sizes, 2020-21 data were unavailable, and 2022-23 data showed lower average costs of €4,314 per stay, with ICU stays averaging €8,351. RSV-adjusted hospitalization costs at the national level ranged from €27.2 million in 2021-22 to €75.6 million in 2018-19.

For high-risk patients aged 65-74, unadjusted RSV-coded costs per stay were higher, spanning from €8,715 to €8,239, peaking at €9,224 in 2019–2020. Unadjusted RSV-coded ICU costs followed a similar pattern, varying from €13,116 to €13,664, with a peak of €14,209 in 2019–2020. RSV-adjusted hospitalization costs at the national level for this group ranged from €8.2 million to €16.5 million (Table 3).

**Table 3.** Economic Burden of RSV-Associated Hospitalization per Season^a^ for Patients Aged over 65 Years (in 2022 Euros).

	2017–2018		2018–2019		2019–2020		2020–20021		2021–2022		July–Dec 2022	
	*N*	Mean (SD)	*N*	Mean (SD)	*N*	Mean (SD)	*N*	Mean (SD)	N	Mean (SD)	*N*	Mean (SD)
**Estimates from two hospitals**												
**Age ≥75 years**												
Average cost per stay, Mean (SD)	1,662	**5,618** (**4,655**)	2,776	5,606 (4,950)	2,158	5,706 (5,117)	–	–	1,355	5,021 (3,239)	3,445	4,314 (3,045)
Broken down by ICU												
with ICU	307	10,482 (8,197)	507	10,938 (8,953)	395	11,127 (9,339)	–	–	164	9,536 (5,511)	402	8,351 (5,042)
without ICU	1,355	4,516 (2,194)	2,269	4,414 (2,077)	1,763	4,491 (2,118)	–	–	1,191	4,399 (2,141)	3,043	3,780 (2,170)
Broken down by GHS and other component												
GHS Cost^b^	1,662	4,780 (2,359)	2,776	4,732 (2,356)	2,158	4,833 (2,539)	–	–	1,355	4,576 (2,238)	3,445	3,960 (2,195)
Additional cost for intensive care	116	1,769 (1,570)	167	2,085 (1,856)	135	2,139 (1,884)	–	–	64	2,443 (2,213)	145	1,979 (2,081)
Additional cost for continuous monitoring	97	1,792 (1,449)	173	1,979 (1,985)	128	2,311 (5,959)	–	–	58	2,463 (2,251)	145	1,823 (1,538)
Additional cost for resuscitation	121	8,380 (8,990)	220	7,883 (9,149)	168	7,725 (7,879)	–	–	48	6,347 (5,657)	110	6,073 (4,885)
Total additional cost for ICU^c^	283	4,922 (7,020)	476	5,094 (7,533)	362	5,200 (7,931)	–	–	146	4,136 (4,396)	364	3,350 (3,748)
**Age 65–74, with chronic conditions**												
Average cost per stay, Mean (SD)	274	8,715 (8,871)	415	8,186 (8,186)	321	9,224 (9,169)	**–**	**–**	248	8,239 (10,449)	532	**6,731 (5,935)**
Broken down by ICU												
with ICU	138	13,116 (10,690)	189	12,772 (9,932)	157	14,209 (10,689)	–	–	107	13,664 (13,769)	213	11,145 (7,088)
without ICU	136	4,249 (1,818)	226	4,351 (2,904)	164	4,452 (2,972)	–	–	141	4,122 (3,084)	319	3,783 (1,883)
Broken down by GHS and other component												
GHS Cost^b^	274	5,250 (2,595)	415	5,330 (3,088)	321	5,730 (3,598)	–	–	248	5,263 (5,460)	532	4,689 (2,584)
Additional cost for intensive care	35	2,786 (3,483)	62	3,064 (5,296)	44	2,884 (6,530)	–	–	33	2,577 (2,923)	58	2,152 (1,768)
Additional cost for continuous monitoring	46	2,733 (3,836)	54	1,868 (1,615)	58	2,159 (2,655)	–	–	32	1,823 (1,432)	58	1,763 (1,472)
Additional cost for resuscitation	77	9,433 (9,871)	111	8,057 (8,148)	93	9,349 (8,100)	–	–	62	9,593 (11,480)	108	7,956 (6,615)
Total additional cost for ICU^c^	129	7,361 (9,394)	184	6,441 (8,833)	146	7,682 (8,370)	–	–	100	7,381 (9,969)	202	5,378 (5,851)
**National Estimates**												
Age 75+, unadjusted	1,662	9,337,279	2,776	15,560,953	2,158	12,312,493	–	–	1,355	6,803,691	3,445	14,860,268
**Age 75+, adjusted by CFRSV**	**8,676**	**48,742,619**	**13,491**	**75,624,214**	**10,466**	**59,713,879**	**–**	**–**	**5,420**	**27,214,764**	**12,681**	**54,698,489**
Age 65–74, with chronic conditions, unadjusted	274	2,387,895	415	3,397,154	321	2,960,968	–	–	248	2,043,282	532	3,580,700
** Age 65–74, with chronic conditions, adjusted by CFRSV**	**1,430**	**12,462,372**	**2,017**	**16,510,987**	**1,557**	**14,362,078**	**–**	**–**	**992**	**8,173,128**	**1,958**	**13,178,591**

Abbreviations: ICU, intensive or continuous care or resuscitation; GHS, stands for “Groupement Homogène de Séjour” which can be translated as Homogenous Stay Group. It is a nationally fixed tariff applied to each GHM (“Groupement Homogène de malade” = diagnosis-related group type of classification); CFRSV: Correction factor for RSV.

^a^Each study season is July of the current year to June next year.

^b^Allocation of 2022 GHS costs, except for 13 hospitalizations with missing data, which were caught up with the price for the corresponding year.

^c^Some RSV hospitalizations with ICU stay do not have ICU supplements in the database.

## DISCUSSION

### Primary Findings

This study estimates the corrected incidence and burden of RSV hospitalizations among adults aged 65 and older in France using data from PMSI. The analysis focused on two key populations: adults aged 75 and older, and those aged 65-74 with chronic conditions (COPD and/or CHF), aligning with French HAS vaccination guidance. A correction factor CFRSV was applied to account for under-reporting, revealing that 75%–80% of RSV-related hospitalizations seemed not captured in national data. After adjustment, the estimated incidence of RSV hospitalizations from 2017 to 2022 ranged from 85 to 221 per 100,000 for those aged 75+ and 161–735 per 100,000 for high-risk individuals aged 65–74. A sensitivity analysis using a broader cardiorespiratory definition further increased the estimated incidence, highlighting additional under-detection in older adults.

### Alignment With Scientific Literature

Our incidence estimates are consistent with other studies in France and internationally. Two studies accounted for under-reporting through modeling approach. Osei-Yeboah et al. estimated an average annual incidence of 190 per 100,000 adults aged 75–84 and 301 per 100,000 for those aged 85 and older in France [31]. Similarly, another PMSI-based study estimated RSV-related hospitalizations for those aged 75+ at 256 per 100,000 [25]. A recent meta-analysis of high-income countries reported laboratory-confirmed RSV hospitalization rates of 100–150 per 100,000 for adults aged 65 and older [32], closely aligning with our findings (Supplementary Table 2).

Additionally, inpatient mortality rates associated with RSV hospitalizations were significant, especially for adults aged 75 and older. In this group, inpatient mortality ranged from 8.9% to 10.4% during the RSV hospitalizations and from 4.2% to 5.4% within 3 months (plus all deaths at home that could not be identified), reflecting the virus's severe impact on this vulnerable population. These findings align with previous studies showing mortality rates of 9.1% for those 75+ [25], 8.0% for those aged 60+ [33], and 7.1% for adults 65+ [32]. Additionally, 4.2%–5.4% of patients aged 75 and older died within 90 days post-discharge, similar to another French study reporting 6.3% mortality within 60 days for adults 60 and older [33]. The 65–74 age group with high-risk comorbidities also showed high mortality (6.0%–9.0%).

Furthermore, the economic burden of RSV hospitalizations in France was substantial. For those aged 75 and older, annual costs ranged from €27.2 million to €75.6 million between 2017 and 2022, with ICU stays contributing heavily. This is consistent with another PMSI study that reported €73.9 million in costs from 2010 to 2020 [25]. The 65–74 age group with comorbidities, though smaller, had disproportionately higher individual costs due to their increased need for intensive care. Depending on the season, the cost of hospitalizations with ICU stays accounted for 66.3%–75.8% of the total costs among high-risk patients aged 65–74 years.

### Clinical and Public Health Importance

These findings show that the burden of RSV on healthcare systems, particularly among the elderly, is greater than previously documented, largely due to under-reporting. Limited RT-PCR access and reliance on less sensitive tests in primary care underestimates RSV incidence [34]. RSV testing is often skipped for adults population, especially outside ICUs. While guidelines from the French Society for Microbiology and the U.S. Centers for Disease Control and Prevention (CDC) recommend expanded molecular testing [18, 19], this alone may not capture the true burden. Several studies suggest that combining serology or sputum samples with RT-PCR testing could increase detection rates by 1.5–2.2 times [6, 35], emphasizing that current estimates may continue to underrepresent RSV's true burden in elderly populations.

The COVID-19 pandemic also affected respiratory virus transmission, including RSV, with a sharp drop in hospitalizations during 2020-21 due to public health measures. The rebound in RSV cases in early 2022-23 reflects increased social interaction, emphasizing the importance of ongoing surveillance. The French Public Health Agency's integrated approach to monitoring Acute Respiratory Infections (ARI) aims to better assess their healthcare impact [36].

Under-reporting of RSV hospitalizations in adults aged 65 and older highlights the need for improved awareness, diagnosis, and coding in this population. Studies show strong evidence of possible causal attribution for RSV following respiratory symptoms (RSV attributable fraction: 88%), with rare co-infections, confirming its role as a primary cause [37]. Unlike in pediatric cases, co-infections are rare in adults, further reinforcing this etiologic role of RSV as a primary cause in respiratory symptoms [38]. Growing evidence shows the impact of RSV on exacerbating comorbidities, leading to cardiorespiratory complications and acute functional decline, which can become prolonged. Consequently, routine screening for RSV in older adults could significantly enhance the accuracy of disease burden estimates and facilitate more targeted interventions.

The high incidence and economic burden of RSV hospitalizations among high-risk adults aged 65–74 suggest the need for preventive measures like vaccination to reduce hospitalizations and healthcare costs. The recent development of RSV vaccines for older adults presents an opportunity to mitigate the virus's impact on healthcare systems.

### Limitations and Strengths

A major strength of this study is its use of France's nationwide hospitalization database, which yields robust national estimates of RSV admissions and costs. Accuracy is further improved by applying an under-reporting correction factor derived from virological data—an approach also used in Denmark [39]—and by stratifying results by age and comorbidity to identify the populations most affected.

Several limitations should be acknowledged. Virological data came from only two Paris hospitals that together represent about 2% of national RSV hospitalizations. Their patients were slightly younger and more likely to require ICU care, so extrapolating directly from these hospitals would exaggerate average severity and expenditure. Population denominators for these hospitals were also unavailable. We therefore applied their correction factors (CFRSV) to the nationwide PMSI dataset, capturing milder, lower-cost cases and delivering a conservative, more balanced yet generalizable estimate.

Diagnostic and coding heterogeneity may cause additional under-reporting of RSV. PCR sensitivity varies with sample type, collection timing, assay, and lower viral loads typical in older adults, while inconsistent ICD-10 coding can mask further cases. We attempted to derive age- and ward-specific correction factors, but small cell sizes required collapsing some strata. However, both hospitals have implemented systematic RSV testing protocols for many years, consistently using high-sensitivity PCR assays, thus supporting CFRSV validity. The absence of exact admission dates in PMSI before 2020 may slightly inflate counts, and analysis of comorbidities only captures individuals with relevant hospitalization records, relying on CNAM data for population estimates, potentially underestimating the incidence of RSV hospitalizations in conditions such as COPD.

Finally, an exploratory sensitivity analysis that broadened the case definition to cardiorespiratory admissions without RSV codes suggests an additional hidden burden—particularly among older adults with cardiorespiratory disease who were rarely tested. Because many such patients receive non-RSV discharge codes, our primary estimates are probably conservative. Larger multi-center datasets with systematic testing across diagnostic categories and seasons will be required to quantify this residual burden more precisely.

### Further Research

Future studies should prioritize nationwide virology-linked real-time RSV surveillance in high-risk adults (e.g., Orchid Consortium (More information about the Orchid project from https://www.santepubliquefrance.fr/projet-orchidee-organisation-d-un-reseau-de-centres-hospitaliers-impliques-dans-la-surveillanceepidemiologique-et-la-reponse-aux-emergences)) for more granular adjustment of the under-reporting. Routine diagnostic testing during peak seasons in hospital settings would provide more accurate data on disease burden and improve public health responses. These efforts should also explicitly include broader high-risk subgroups—such as immunocompromised adults and those with chronic conditions like diabetes or hypertension—to inform any future expansion of vaccination recommendations. Additionally, there is a need to better understand post-hospitalization outcomes, as this study highlights significant mortality and rehospitalization rates. Investigating long-term outcomes is essential for guiding clinical care and improving patient management.

Research should also evaluate the long-term impact of RSV vaccines on reducing hospitalizations, mortality, and healthcare costs among older adults. From a policy perspective, implementing vaccination programs for high-risk adults could reduce RSV hospitalizations. Raising awareness among healthcare providers about the role of RSV, the importance of screening, and improving coding accuracy could further enhance national RSV data accuracy.

## CONCLUSIONS

In summary, we found that RSV poses a significant and underreported burden on elderly populations in a high-income country, especially those with chronic conditions such as COPD and CHF. By accounting for under-reporting, we showed that a substantial proportion of RSV-related hospitalizations, ICU admissions, and deaths remain undetected in national health data. The findings highlight the need for better diagnostic practices, increased awareness, and preventive measures to reduce the health and economic impact of RSV on high-risk older adults. Improved surveillance and accurate coding of RSV cases are essential for informing public health policy and enhancing patient care.

## Supplementary Material

ofaf528_Supplementary_Data

## Supplementary Data

Supplementary materials are available at *Open Forum Infectious Diseases* online. Consisting of data provided by the authors to benefit the reader, the posted materials are not copyedited and are the sole responsibility of the authors, so questions or comments should be addressed to the corresponding author.
