# Supplementary material for: Incidence and Burden of Respiratory Syncytial Virus-Associated Hospitalizations Among People 65 and Older in France: A National Hospital Database Study
Source: Open Forum Infect Dis. 2025 Sep 15;12(9):ofaf528. doi: 10.1093/ofid/ofaf528 (PMC12453078; doi:10.1093/ofid/ofaf528)
Supplement: ofaf528_Supplementary_Data [file ofaf528_supplementary_data.docx]

**Supplementary Materials**

**Supplementary Table 1. Baseline Characteristics Patients with RSV-coded Hospitalizations Recorded in the French National Hospital Discharge Database (PMSI) in Pitié-Bichat Cohort and at National Level over the Study Period**

|  | **07/2017-12/2022** | |
| --- | --- | --- |
|  | **Pitié-Salpêtrière and Bichat Claude Bernard University Hospital** | **National** |
| **N total** | 353 | 20929 |
| **Age (in categories), N (%)** |  |  |
| 18-49 years | 61 (17.3) | 1945 (9.3) |
| 50-59 years | 55 (15.6) | 1775 (8.5) |
| 60-64 years | 46 (13.0) | 1518 (7.3) |
| 65-69 years | 46 (13.0) | 1889 (9.0) |
| 70-79 years | 74 (21.0) | 4506 (21.5) |
| ≥80 years | 71 (20.1) | 9296 (44.4) |
| **Age (in categories), N (%)** |  |  |
| <65 years | 162 (45.9) | 5238 (25.0) |
| ≥ 65 years | 191 (54.1) | 15691 (75.0) |
| **Sex, N (%)** |  |  |
| Male | 169 (47.9) | 9204 (44.0) |
| Female | 184 (52.1) | 11725 (56.0) |
| **At least one stay in ICU (intensive or continuous care or resuscitation), N (%)** |  |  |
| No | 253 (71.7) | 16091 (76.9) |
| Yes | 100 (28.3) | 4838 (23.1) |
| **At least one stay in ICU (intensive or continuous care), N (%)** |  |  |
| No | 293 (83.0) | 17823 (85.2) |
| Yes | 60 (17.0) | 3106 (14.8) |
|  |  |  |

**ALT-TEXT:** Supplementary Table 1 presents baseline characteristics of patients with RSV-coded hospitalizations recorded in the French National Hospital Discharge Database (PMSI) between July 2017 and December 2022, at the national level and specifically for the two Paris hospitals: Pitié-Salpêtrière and Bichat. Variables include age group, sex, and ICU admission status. The data from the two hospitals reflect a higher proportion of younger patients and severe cases.

**Supplementary Table 2. Incidence of RSV Hospitalizations at National level, Unadjusted and Adjusted by Correction Factor (CFRSV), Age 65 and Older**

|  | **Age >=65 Years** | |
| --- | --- | --- |
| **Study Season**^a^ | **Reported from PMSI** | **Adjusted by CFRSV** |
| **Number of RSV Hospitalizations in France** |  |  |
| 2017-18 | 2,235 | 11,667 |
| 2018-19 | 3,669 | 17,831 |
| 2019-20 | 2,888 | 14,007 |
| 2020-21 | NA | NA |
| 2021-22 | 1,902 | 7,608 |
| July-Dec 22 | 4,698 | 17,289 |
|  |  |  |
| **Incidence of RSV hospitalization in France, (per 100,000 adult residents)** | |  |
| 2017-18 | 17.52 | 91.44 |
| 2018-19 | 28.14 | 136.75 |
| 2019-20 | 21.69 | 105.19 |
| 2020-21 | NA | NA |
| 2021-22 | 13.79 | 55.17 |
| July-Dec 22 | 33.78 | 124.31 |
| Abbreviations: CFRSV, correction factor for RSV |  |  |
| ^a^Each study season is July of current year to June next year |  |  |

**ALT-TEXT: S**upplementary Table 2 presents the number and incidence of respiratory syncytial virus (RSV) hospitalizations in France among adults aged 65 years and older, across six study seasons. For each season from 2017–18 to July–December 2022, both the number of hospitalization cases reported from the PMSI database and those adjusted using a correction factor for RSV (CFRSV) are shown. Incidence rates per 100,000 population are provided for both unadjusted and adjusted figures. Data for the 2020–21 season are not available due to the absence of a significant RSV epidemic during that period. The table highlights the substantial increase in estimated RSV burden after adjustment, particularly during the 2018–19 and July–December 2022 seasons.

| **Supplementary Table 3. Estimated Number of RSV Hospitalizations at National Level, Adjusted Using Cardiorespiratory Main Diagnosis, Age 65 and Older (Exploratory Analysis)** | | | | | | |
| --- | --- | --- | --- | --- | --- | --- |
|  | **2017-18** | **2018-19** | **2019-20** | **2020-21^2^** | **2021-22** | **July - Dec 2022** |
|  |  |  |  |  |  |  |
| Correction factor (CFRSV) derived using data from Pitié / Bichat hospitals - all units ^1^ | 5.22 | 4.86 | 4.85 | NA | 4.00 | 3.68 |
| Number of **RSV** hospitalizations with **CR** main diagnosis in Pitié / Bichat hospitals reported in PMSI | 38 | 47 | 37 | ≤ 10 | 21 | 27 |
| Adjusted number of **RSV** hospitalizations with **CR** main diagnosis after application of CFRSV in Pitié / Bichat hospitals | 198 | 228 | 179 | NA | 84 | 99 |
| Total number of hospitalizations with **CR** main diagnosis in Pitié / Bichat hospitals reported in PMSI | 14,172 | 13,895 | 11,816 | 11,645 | 12,427 | 5,999 |
| Proportion of **RSV** hospitalizations among **CR** hospitalizations (main diagnosis) in Pitié / Bichat hospitals (CF_CR_)^3^ | 0.014 | 0.016 | 0.015 | NA | 0.007 | 0.017 |
| Total number of **CR** hospitalizations (main diagnosis) at national level reported in PMSI | 1,475,642 | 1,491,975 | 1,372,769 | NA | 1,385,181 | 685,947 |
| **Estimated number of RSV hospitalizations among CR hospitalizations (main diagnosis) using CF_CR_** | **20,654** | **24,527** | **20,848** | **NA** | **9,363** | **11,361** |
| ^1^ Correction factor (CFRSV)= Number of RSV positive tests in Pitié-Salpêtrière and Bichat Claude Bernard University hospitals divided by the number of RSV coded-hospitalizations from the PMSI in these two hospitals; presentation of these age groups across all-units due to small sample sizes. | | | | | | |
| ^2^ Season 2020-2021 is not presented because the sample size is too small. | |  |  |  |  |  |
| ^3^CF_CR_= Adjusted Nb of RSV hospitalization with cardio-respiratory main diagnosis hospitalizations at Pitié - Bichat / Nb of cardio-respiratory hospitalizations (CR as main diagnosis) at Pitié - Bichat, for example, in 2017-18 season, 198/14,172=0.014 | | | | | | |

**Methodology Overview:**

To capture RSV cases hidden under broad cardiac or respiratory (CR) diagnoses, we created a second correction factor, **CF_CR_**. The steps were:

- Identify candidate cases – Count admissions at Pitié-Salpêtrière and Bichat Claude Bernard University Hospitals with RSV code with CR as primary diagnosis in PMSI.
- Adjust for RSV under-coding – Multiply this count by the original RSV correction factor (**CFRSV**) to correct for under-reporting.
- Derive **CF_CR_** – Divide the adjusted RSV count by the total CR primary-diagnosis admissions in the two hospitals. This proportion is **CF_CR._**
- Apply nationally – Multiply **CF_CR_** by the national total of CR primary-diagnosis admissions in PMSI to estimate RSV-related CR hospitalizations across France.

This approach accounts for both under-reporting and potential under-diagnosis of RSV in adults with cardiac or respiratory presentations.

**ALT-TEXT:** Supplementary Table 3 presents an exploratory analysis estimating RSV hospitalizations in adults aged 65 and older in France between 2017 and December 2022. Data from the PMSI database were adjusted for under-diagnosis of RSV among hospitalizations with a primary cardiorespiratory diagnosis, based on correction factors derived from two Paris hospitals (Pitié-Salpêtrière and Bichat). The table reports annual correction factors (CFRSV and CFCR), intermediate calculations, and estimated national RSV hospitalization counts in this subgroup."
